# Ultra‐Early Recurrence of Atrial Fibrillation After Direct Cardioversion Predicts Late Recurrence After Ablation for Persistent Atrial Fibrillation

**DOI:** 10.1002/joa3.70212

**Published:** 2025-10-17

**Authors:** Hiroto Sugiyama, Yoshimi Onishi, Tatsuya Onuki, Shuhei Arai, Kosuke Yoshikawa, Hiroshi Mase, Yuya Nakamura, Masaaki Kurata, Yoshitaka Iso, Taku Asano, Hiroshi Suzuki, Toshiro Shinke

**Affiliations:** ^1^ Division of Cardiology, Department of Medicine Showa Medical University Tokyo Japan; ^2^ Division of Cardiology, Department of Medicine Showa Medical University Fujigaoka Hospital Yokohama Japan

**Keywords:** catheter ablation, immediate recurrence of atrial fibrillation, persistent atrial fibrillation, ultra‐early recurrence of atrial fibrillation

## Abstract

**Background:**

Although immediate recurrence of atrial fibrillation (IRAF) after cardioversion has been proposed as a surrogate for atrial substrate vulnerability, its broad definition may insufficiently discriminate patients at highest risk of postablation recurrence. We introduced the concept of ultra‐early recurrence of AF (URAF)—defined as recurrence within 10 s after direct current cardioversion (CV) under deep sedation—as a novel marker of advanced remodeling in persistent atrial fibrillation (AF).

**Objective:**

To evaluate whether URAF independently predicts late recurrence following pulmonary vein isolation (PVI) in patients with persistent or long‐standing persistent AF.

**Methods:**

We retrospectively analyzed 104 patients undergoing first‐time PVI for persistent AF. Among 93 patients who underwent external CV, URAF, and IRAF were defined as AF recurrence within 10 and 90 s, respectively. Recurrence of atrial arrhythmias was assessed at 12 m postablation.

**Results:**

URAF was observed in 10/104 patients (9.6%) and was associated with higher 12‐m recurrence compared with URAF‐negative patients (50% vs. 18%, *p* = 0.02), whereas IRAF (20/104, 19.2%) showed no significant difference (30% vs. 19%, *p* = 0.28). On multivariable logistic regression, URAF (Odds Ratio (OR): 4.8; 95% Confidence Interval (CI): 1.16–19.98; *p* = 0.029) and long‐standing AF (OR: 5.5; 95% CI: 1.70–17.78; *p* = 0.004) emerged as independent predictors of recurrence. Kaplan–Meier analysis showed worse recurrence‐free survival for URAF (log‐rank *p* = 0.02; HR 4.5, 95% CI 1.18–17.41).

**Conclusion:**

URAF may represent a promising intra‐procedural marker associated with post‐ablation recurrence in persistent AF, but prospective validation in larger cohorts is required.

## Graphical abstract

Ultra‐early recurrence of AF (URAF) within 10 s after cardioversion predicted late recurrence after PVI in persistent AF. (OR: 4.8; 95% CI: 1.16–19.98; *p* = 0.029). URAF may represent a promising intraprocedural marker associated with postablation recurrence in persistent AF.

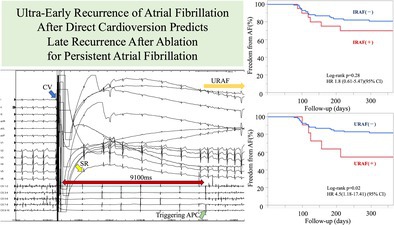

## 1 Introduction

Pulmonary vein isolation (PVI) is a cornerstone of catheter ablation for atrial fibrillation (AF). However, its efficacy in patients with persistent or long‐standing persistent AF remains suboptimal despite complete procedural success [1, 2, 3]. This limited effectiveness is primarily attributed to the presence of an arrhythmogenic substrate that extends beyond pulmonary vein (PV) triggers, particularly in the setting of advanced atrial remodeling [4, 5].

In addition to PV ectopy, non‐PV premature atrial contractions, structural fibrosis, and altered autonomic tone are recognized contributors to AF maintenance [6, 7]. Because the degree of substrate remodeling is difficult to quantify in real‐time clinical settings, a procedural marker that reflects the underlying atrial vulnerability would be of significant clinical utility.

There is a phenomenon called immediate recurrence of AF (IRAF), which is a reproducible recurrence of AF immediately after electric cardioversion (CV) and restoration of sinus rhythm (SR) [8, 9].

IRAF following CV has emerged as a potential surrogate for such remodeling. While previous studies have defined IRAF as AF recurrence within 90 s, this broad definition may encompass heterogeneous mechanisms and insufficiently discriminate high‐risk substrates [10]. Inoue et al. reported that, in patients with persistent AF, IRAF was associated with late AF recurrence unless the initiating trigger was successfully ablated, highlighting the importance of trigger‐substrate interaction in procedural outcome [11].

In this context, we introduce the concept of ultra‐early recurrence of AF (URAF)—defined as AF recurrence within 10 s after external CV under deep sedation. This narrow time frame occurs in a parasympathetic‐dominant state, reducing confounding from sympathetic activation [12], and may more accurately reflect intrinsic substrate vulnerability.

We hypothesized that URAF—occurring within a short, parasympathetic‐dominant window under deep sedation—may serve as a real‐time biomarker of atrial vulnerability, with stronger predictive value than conventional IRAF. This study was designed as an exploratory, hypothesis‐generating investigation.

## 2 Methods

### 2.1 Study Population

We retrospectively reviewed the medical records of consecutive patients who underwent first‐time catheter ablation for persistent or long‐standing AF at Showa Medical University Fujigaoka Hospital between January 2019 and September 2023. Persistent AF was defined as sustained AF lasting longer than 7 days but less than 1 year; long‐standing persistent AF was defined as AF lasting for more than 1 year [13].

All patients underwent detailed clinical evaluation, including physical examination, laboratory testing, 12‐lead electrocardiography (ECG), transthoracic echocardiography, and electrophysiological study. The study complied with the Declaration of Helsinki and was approved by the institutional ethics committee (Approval number: 2023‐195‐A). All patients provided informed consent.

### 2.2 Preprocedural Management

All patients were anticoagulated for ≥ 1 month before the procedure. Antiarrhythmic drugs (AADs) were discontinued for at least five half‐lives prior to ablation.

### 2.3 Ablation Procedure

All procedures were performed under deep sedation using dexmedetomidine and propofol in combination, with a target bispectral index < 60 and controlled ventilation via a laryngeal mask airway. Real‐time esophageal temperature monitoring was employed throughout the procedure. A decapolar catheter was positioned in the coronary sinus via femoral access. Transseptal puncture was guided by intracardiac echocardiography.

Left atrial (LA) electroanatomical mapping was performed using either the EnSite NavX system (Abbott Cardiovascular, Abbott Park, IL) or CARTO 3 system (Biosense Webster Inc., Irvine, California). A high‐density mapping catheter (HD Grid or Pentaray) was used in combination with an irrigated contact force‐sensing ablation catheter in a bidirectional steerable sheath.

All pulmonary vein isolations were performed using an irrigated ablation catheter in a point‐by‐point fashion, delivering 50 W for 10 s per lesion. The temperature was limited to 43°C. PVI was performed with the endpoint of bidirectional block. Additional ablation lines, including LA posterior wall or cavotricuspid isthmus ablation, were delivered at the operator's discretion. No artificial AF induction (e.g., burst pacing or extra‐atrial stimulation) was performed after PVI.

### 2.4 Direct Current Cardioversion and AF Recurrence Classification

For patients presenting in AF, synchronized external CV was administered immediately before ablation using an initial energy of 100 J. If AF persisted, stepwise escalation up to 270 J was employed. To minimize mechanically induced atrial ectopy during the vulnerable postcardioversion period, catheters positioned in the left atrium were intentionally kept stationary and away from the endocardial surface. No catheter manipulation was performed during the 90 s following CV. During this 90‐s observation, no catheter was positioned in the right atrium; left‐atrial mapping catheters were deliberately kept off the endocardium (floating), and continuous reference recordings were obtained only from a decapolar catheter in the coronary sinus. Accordingly, exact initiation sites were not systematically localized. For measurement of AF‐recurrence timing after cardioversion, only CV episodes with successful AF termination and documentation of at least one sinus beat on intracardiac electrograms were included.

After successful cardioversion, recurrence of AF was continuously monitored using intracardiac electrograms. Sustained AF was defined as recurrence lasting > 30 s. URAF was defined as AF recurrence within 10 s of successful CV, based on continuous intraprocedural electrogram monitoring. The prespecified ≤ 10‐s cut‐off was chosen to capture events within a parasympathetic‐dominant window under deep sedation while all catheters were kept immobile for 90 s, thereby minimizing confounding from sympathetic activation and catheter manipulation and enriching for substrate‐related vulnerability. In post hoc sensitivity analyses, alternative thresholds (≤ 15,≤ 30, and≤ 60 s) were evaluated using the same analytic framework; results are summarized in Table 2. IRAF was defined as recurrence within 90 s, in accordance with prior literature [10]. The coupling interval of the triggering premature atrial contraction (APC) was measured on the coronary‐sinus reference as the interval from the preceding sinus QRS (or last organized atrial deflection) to the APC initiating AF.

Both URAF and IRAF were assessed in real time and documented as binary variables (present/absent) based on electrogram findings. The time from CV to the occurrence of AF was evaluated. We defined AF occurrence as > 30 s of sustained AF. We did not perform intraprocedural cardioversion in patients who had undergone external cardioversion before the procedure and remained in sinus rhythm on arrival; these patients were classified as IRAF−/URAF− in timing‐based analyses.

### 2.5 Follow‐Up Protocol

Patients were followed at 3, 6, and 12 months with 12‐lead ECG and 24‐hour Holter monitoring. AF recurrence was defined as atrial arrhythmias (AF, flutter, or tachycardia) lasting > 30 s beyond a 90‐day blanking period. Patients who did not complete 12‐month follow‐up were excluded.

### 2.6 Statistical Analysis

Continuous variables are presented as mean ± standard deviation, and categorical variables as count (percentage). Group comparisons were conducted using the Mann–Whitney U test for continuous variables and the chi‐square test for categorical variables. Statistical significance was defined as *p* < 0.05.

Univariate and multivariable logistic regression analyses were performed to identify independent predictors of AF recurrence. Odds ratios (OR) and 95% confidence intervals (CI) were reported. Kaplan–Meier analysis with log‐rank testing was used to compare time‐to‐recurrence. All analyses were conducted using JMP version 16.0 (SAS Institute, Cary, NC, USA).

### 2.7 Redo Assessment and Statistics

At redo procedures, the prespecified procedural assessment was limited to identification of pulmonary vein (PV) reconnection; other mechanisms were not systematically mapped. Differences in reconnection proportions by URAF status (URAF− vs. URAF+) and IRAF status (IRAF− vs. IRAF+) were evaluated using Fisher's exact test (two‐sided).

## 3 Results

We reviewed the medical records of 105 consecutive patients who underwent PVI for persistent AF between January 2019 and September 2023. One patient was excluded due to incomplete follow‐up after transfer to another institution, leaving 104 patients in the final analysis. Baseline characteristics are summarized in Table 1. The mean age was 68.0 ± 9.8 years (range: 37–85), with 69 patients (66%) being male. The most common comorbidity was hypertension (54%). Twenty‐two patients (21%) had AF diagnosed ≥ 2 years prior, and 16 patients (15%) met the criteria for long‐standing persistent AF. Left ventricular ejection fraction < 50% was present in 29 patients (28%), and LA diameter > 40 mm in 61 patients (59%). Fourteen patients (14%) had a history of hospitalization for heart failure. The mean CHADS2 score was 1.8 ± 1.2, and the mean BNP level was 165.9 ± 148.1 pg/mL. None of the patients resumed AADs throughout the follow‐up period.

**TABLE 1:** Baseline characteristics of the study population.

Characteristics	Total 104 (100%)	Recurrence (−) 82 (78.8%)	Recurrence (+) 22 (21.2%)	*p*
Male, *n* (%)	69 (66%)	58 (71%)	11 (50%)	0.06
Age > 75, *n* (%)	25 (24%)	17 (21%)	8 (36%)	0.13
Hypertension, *n* (%)	56 (54%)	42 (51%)	14 (64%)	0.30
Dyslipidemia, *n* (%)	39 (38%)	33 (40%)	6 (27%)	0.26
Diabetes, *n* (%)	22 (21%)	19 (23%)	3 (14%)	0.33
Ejection fraction < 50%, *n* (%)	29 (28%)	23 (28%)	6 (27%)	0.94
LA diameter > 40 mm, *n* (%)	61 (59%)	48 (59%)	13 (59%)	0.96
Obesity (BMI ≥ 25), *n* (%)	35 (34%)	31 (38%)	4 (18%)	0.08
Alcohol > 140 g, *n* (%)	13 (13%)	11 (13%)	2 (9%)	0.59
Smoking, *n* (%)	49 (47%)	40 (49%)	9 (41%)	0.51
Chronic kidney disease, *n* (%)	49 (47%)	40 (49%)	9 (41%)	0.51
Hospitalization for heart failure, *n* (%)	14 (14%)	10 (12%)	4 (18%)	0.47
BNP, pg/mL	165.9 ± 148.1	163.4 ± 151.4	175.5 ± 141.7	0.73
CHADS2 score	1.8 ± 1.2	1.7 ± 1.2	2.1 ± 1.2	0.16
β‐blocker, *n* (%)	72 (69%)	56 (68%)	16 (73%)	0.69
ACE‐I/ARB, *n* (%)	55 (53%)	41 (50%)	14 (64%)	0.26
Diuretic, *n* (%)	32 (31%)	26 (32%)	6 (27%)	0.69
Period from first diagnosis > 2 y, *n* (%)	22 (21%)	14 (17%)	8 (36%)	0.05
Long‐standing AF, *n* (%)	16 (15%)	8 (10%)	8 (36%)	0.002

Abbreviations: ACE‐I, angiotensin converting enzyme inhibitor; AF, atrial fibrillation; ARB, angiotensin receptor blocker; BMI, body mass index; BNP: brain natriuretic peptide; LA, left atrial.

11 patients (11%) maintained SR before the procedure using AADs or preadmission CV and did not undergo intraprocedural CV. The remaining 93 patients (89%) underwent external CV under deep sedation prior to LA mapping or ablation.

**FIGURE 1:**
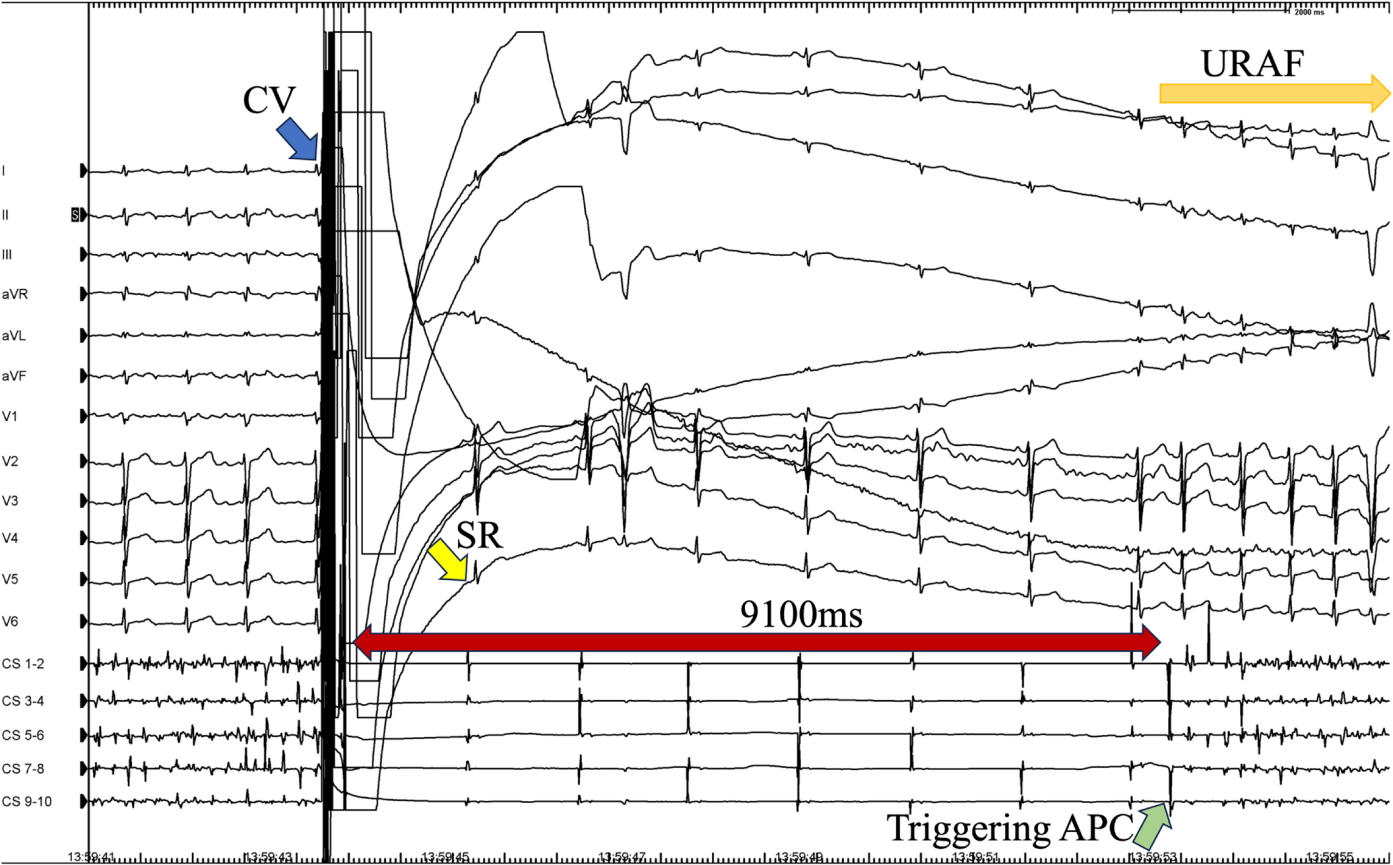
Representative case of ultra‐early recurrence of AF. Surface electrocardiogram and intracardiac electrograms during pulmonary vein isolation showing ultra‐early recurrence of atrial fibrillation, defined as AF recurrence within 10 s following successful CV under deep sedation. URAF: Ultra‐early recurrence of atrial fibrillation; AF: Atrial fibrillation; CV: Direct current cardioversion.

Among the 93 patients who received CV, AF recurred during the procedure in 35 patients (38%). The average number of shocks was 1.1 ± 0.6, with a mean energy of 130 ± 45.4 J. Ten patients (9.6%) demonstrated URAF, defined as recurrence within 10 s (Figure 1). Twenty patients (19%) met the definition of IRAF, defined as recurrence within 90 s. All URAF patients were also included in the IRAF group. The mean time to IRAF was 18.2 ± 19.6 s.

All patients achieved successful PVI. Additional ablation was performed at the LA posterior wall in 18 patients (17%) and at the cavotricuspid isthmus in 37 patients (36%). The mean number of CV shocks required for successful cardioversion was 1.1 ± 0.6, and the mean delivered energy was 130 ± 45 J; neither parameter differed significantly between patients with and without recurrence. All CV attempts used for recurrence‐timing assessment achieved AF termination with documentation of at least one sinus beat; no cases lacked sinus restoration. Furthermore, no ablation targeting premature atrial contractions presumed to trigger IRAF or URAF was performed in any case. Procedural characteristics are summarized in Table 2.

**TABLE 2:** Procedural characteristics.

Characteristics	Total 104 (100%)	Recurrence (−) 82 (78.8%)	Recurrence (+) 22 (21.2%)	*p*
CTI ablation, *n* (%)	37 (36%)	33 (40%)	4 (18%)	0.05
LA posterior wall ablation, *n* (%)	18 (17%)	12 (15%)	6 (27%)	0.16
AF occurrence after CV, *n* (%)	35 (34%)	25 (30%)	10 (45%)	0.32
AF occurrence within 10 s after CV (URAF), *n* (%)	10 (9.6%)	5 (6%)	5 (23%)	0.02
AF occurrence within 15 s after CV, *n* (%)	14 (13%)	9 (11%)	5 (23%)	0.15
AF occurrence within 30 s after CV, *n* (%)	17 (16%)	12 (15%)	5 (23%)	0.36
AF occurrence within 45 s after CV, *n* (%)	18 (17%)	12 (15%)	6 (27%)	0.16
AF occurrence within 60 s after CV, *n* (%)	20 (19%)	14 (17%)	6 (27%)	0.28
AF occurrence within 90 s after CV (IRAF), *n* (%)	20 (19%)	14 (17%)	6 (27%)	0.28
SR maintenance before PVI, *n* (%)	11 (11%)	10 (12%)	1 (4.5%)	0.63
CV energy, J	130 ± 45.4	131 ± 49.2	129 ± 29.2	0.83
Number of CV attempts, *n*	1.1 ± 0.6	1.1 ± 0.6	1.1 ± 0.5	0.64

Abbreviations: AF, atrial fibrillation; CTI, cavotricuspid isthmus; CV, direct current cardioversion; IRAF, immediate recurrence of atrial fibrillation; LA, left atrial; PVI, pulmonary vein isolation; SR, sinus rhythm; URAF; ultra‐early recurrence of atrial fibrillation.

Tables 3 and 4 summarize the comparisons of baseline characteristics and recurrence rates according to the presence or absence of URAF and IRAF, respectively. A significant difference in atrial arrhythmia recurrence was observed between patients with and without URAF (50% vs. 18%, *p* = 0.02), whereas no such difference was seen between those with and without IRAF (30% vs. 19%, *p* = 0.28). Both URAF and IRAF were associated with a higher number of CV times (1.6 ± 0.7 vs. 1.0 ± 0.5, *p* = 0.006, 1.4 ± 0.6 vs. 1.0 ± 0.5, *p* = 0.01) and more frequent use of posterior wall ablation (50% vs. 14%, *p* = 0.004; 35% vs. 13%, *p* = 0.02). Categorical variables were compared using the chi‐square test.

**TABLE 3:** Comparison between patients with and without ultra‐early recurrence of AF.

Characteristics	Total 104 (100%)	URAF (+) 10 (9.6%)	URAF (−) 94 (90.4%)	*p*
Recurrence after PVI, *n* (%)	22 (21%)	5 (50%)	17 (18%)	0.02
Male, *n* (%)	69 (66%)	7 (70%)	62 (66%)	0.80
Age > 75, *n* (%)	25 (24%)	1 (10%)	24 (26%)	0.27
Hypertension, *n* (%)	56 (54%)	3 (30%)	53 (56%)	0.11
Dyslipidemia, *n* (%)	39 (38%)	4 (40%)	35 (37%)	0.86
Diabetes, *n* (%)	22 (21%)	2 (20%)	20 (21%)	0.97
Ejection fraction < 50%, *n* (%)	29 (28%)	1 (10%)	28 (30%)	0.18
LA diameter > 40 mm, *n* (%)	61 (59%)	7 (70%)	54 (58%)	0.44
Obesity (BMI ≥ 25), *n* (%)	35 (34%)	3 (30%)	32 (34%)	0.80
Alcohol > 140 g, *n* (%)	13 (13%)	2 (20%)	11 (12%)	0.45
Smoking, *n* (%)	49 (47%)	7 (70%)	42 (45%)	0.13
Chronic kidney disease, *n* (%)	49 (47%)	3 (30%)	46 (49%)	0.25
Hospitalization for heart failure, *n* (%)	14 (14%)	1 (10%)	13 (14%)	0.74
BNP, pg/mL	165.9 ± 148.1	158.9 ± 114.8	166.7 ± 152.5	0.87
CHADS2 score	1.8 ± 1.2	1.4 ± 1.17	1.8 ± 1.2	0.31
β‐blocker, *n* (%)	72 (69%)	7 (70%)	65 (69%)	0.95
ACE‐I/ARB, *n* (%)	55 (53%)	3 (30%)	52 (55%)	0.12
Diuretic, *n* (%)	32 (31%)	1 (10%)	31 (33%)	0.13
Period from first diagnosis > 2 y, *n* (%)	22 (21%)	2 (20%)	20 (21%)	0.93
Long‐standing AF, *n* (%)	16 (15%)	2 (20%)	14 (15%)	0.67
CTI ablation, *n* (%)	37 (36%)	3 (30%)	34 (36%)	0.70
LA posterior wall ablation, *n* (%)	18 (17%)	5 (50%)	13 (14%)	0.004
Trigger ablation, *n*	0	0	0	N/A
CV energy, J	130 ± 45.4	145 ± 43.8	128.7 ± 45.8	0.29
Number of CV attempts, *n*	1.1 ± 0.6	1.6 ± 0.7	1.0 ± 0.5	0.006

Abbreviations: ACE‐I, angiotensin converting enzyme inhibitor; AF, atrial fibrillation; ARB, angiotensin receptor blocker; BMI, body mass index; BNP, brain natriuretic peptide; CTI, cavotricuspid isthmus; CV, direct current cardioversion.LA, left atrial; PVI, pulmonary vein isolation.

**TABLE 4:** Comparison between patients with and without immediate recurrence of AF.

Characteristics	Total 104 (100%)	IRAF (+) 20 (19.2%)	IRAF (−) 84 (80.8%)	*p*
Recurrence after PVI, *n* (%)	22 (21%)	6 (30%)	16 (19%)	0.28
Male, *n* (%)	69 (66%)	12 (60%)	57 (68%)	0.50
Age > 75, *n* (%)	25 (24%)	2 (10%)	23 (27%)	0.10
Hypertension, *n* (%)	56 (53%)	7 (35%)	49 (58%)	0.06
Dyslipidemia, *n* (%)	39 (38%)	7 (35%)	32 (38%)	0.80
Diabetes, *n* (%)	22 (21%)	4 (20%)	18 (21%)	0.89
Ejection fraction < 50%, *n* (%)	29 (28%)	4 (20%)	25 (30%)	0.38
LA diameter > 40 mm, *n* (%)	61 (59%)	11 (55%)	50 (60%)	0.71
Obesity (BMI ≥ 25), *n* (%)	35 (34%)	5 (25%)	30 (36%)	0.36
Alcohol > 140 g, *n* (%)	13 (13%)	4 (20%)	9 (11%)	0.26
Smoking, *n* (%)	49 (47%)	12 (60%)	37 (44%)	0.20
Chronic kidney disease, *n* (%)	49 (47%)	7 (35%)	42 (50%)	0.23
Hospitalization for heart failure, *n* (%)	14 (13%)	1 (5%)	13 (15%)	0.22
BNP, pg/mL	165.9 ± 148.1	140.4 ± 95.1	172.0 ± 158.8	0.40
CHADS2 score	1.8 ± 1.2	1.3 ± 1.2	1.9 ± 1.2	0.05
β‐blocker, *n* (%)	72 (69%)	13 (65%)	59 (70%)	0.65
ACE‐I/ARB, *n* (%)	55 (53%)	8 (40%)	47 (56%)	0.20
Diuretic, *n* (%)	32 (31%)	4 (20%)	28 (33%)	0.25
Period from first diagnosis > 2 y, *n* (%)	22 (21%)	2 (10%)	20 (24%)	0.17
Long‐standing AF, *n* (%)	16 (15%)	4 (20%)	12 (14%)	0.52
CTI ablation, *n* (%)	37 (36%)	5 (25%)	32 (38%)	0.27
LA posterior wall ablation, *n* (%)	18 (17%)	7 (35%)	11 (13%)	0.02
Trigger ablation, *n*	0	0	0	N/A
CV energy, J	130 ± 45.4	135 ± 40.1	129.2 ± 47.3	0.61
Number of CV attempts, *n*	1.1 ± 0.6	1.4 ± 0.6	1.0 ± 0.5	0.01

Abbreviations: ACE‐I, angiotensin converting enzyme inhibitor; AF, atrial fibrillation; ARB, angiotensin receptor blocker; BMI, body mass index; BNP, brain natriuretic peptide; CTI, cavotricuspid isthmus; CV, direct current cardioversion; LA: left atrial; PVI, pulmonary vein isolation.

Among recurrences within 90 s, the APC coupling interval did not differ significantly between URAF and non‐URAF immediate recurrences (225.6 ± 23.2 ms vs. 206.2 ± 74.0 ms; *p* = 0.256).

During the 12‐month follow‐up after a 90‐day blanking period, 22 patients (21%) experienced clinical AF recurrence. AF duration ≥ 2 years (36% vs. 17%, *p* = 0.05), long‐standing AF (36% vs. 10%, *p* = 0.002) (Table 1), and URAF (23% vs. 6%, *p* = 0.02) were more frequent in the recurrence group than in the nonrecurrence group (Table 2). IRAF was also numerically more frequent in the recurrence group (27% vs. 17%), but this difference did not reach statistical significance (*p* = 0.28). In sensitivity analyses, alternative thresholds (≤ 15, ≤ 30, and ≤ 60 s) did not outperform the prespecified ≤ 10‐s definition; effect estimates were weaker, and none reached statistical significance (Table 2). No significant difference in recurrence was observed between patients who maintained SR before the procedure and those who did not.

In univariate analysis, both URAF (OR: 4.5; 95% CI: 1.18–17.41; *p* = 0.03) and long‐standing AF (OR: 5.3; 95% CI: 1.70–16.4; *p* = 0.005) were significantly associated with recurrence. IRAF did not reach statistical significance in univariate analysis (OR: 1.8; 95% CI: 0.61–5.47; *p* = 0.29).

Multivariable logistic regression identified URAF (OR: 4.8; 95% CI: 1.16–19.98; *p* = 0.029) and long‐standing AF (OR: 5.5; 95% CI: 1.70–17.78; *p* = 0.004) as independent predictors of AF recurrence (Table 5).

**TABLE 5:** Univariate and multivariable analyses for predictors of AF recurrence.

Clinical characteristics	Univariate analysis		Multivariable analysis	
	OR (95% CI)	*p*	OR (95% CI)	*p*
URAF IRAF Age Male Left atrial diameter CHADS2 score Long‐standing AF Period from first diagnosis < 2 years	4.5 (1.18–17.41) 1.8 (0.61–5.47) 1.0 (0.92–1.02) 0.4 (0.16–1.08) 1.0 (0.92–1.09) 0.8 (0.50–1.12) 5.3 (1.70–16.4) 0.4 (0.13–1.02)	0.03 0.28 0.28 0.07 0.99 0.15 0.005 0.05	4.8 (1.16–19.98) 5.5 (1.70–17.78)	0.029 0.004

Abbreviations: AF, atrial fibrillation; CI, confidence interval; IRAF, immediate recurrence of atrial fibrillation; OR, odds ratio; URAF, ultra‐early recurrence of atrial fibrillation.

**FIGURE 2:**
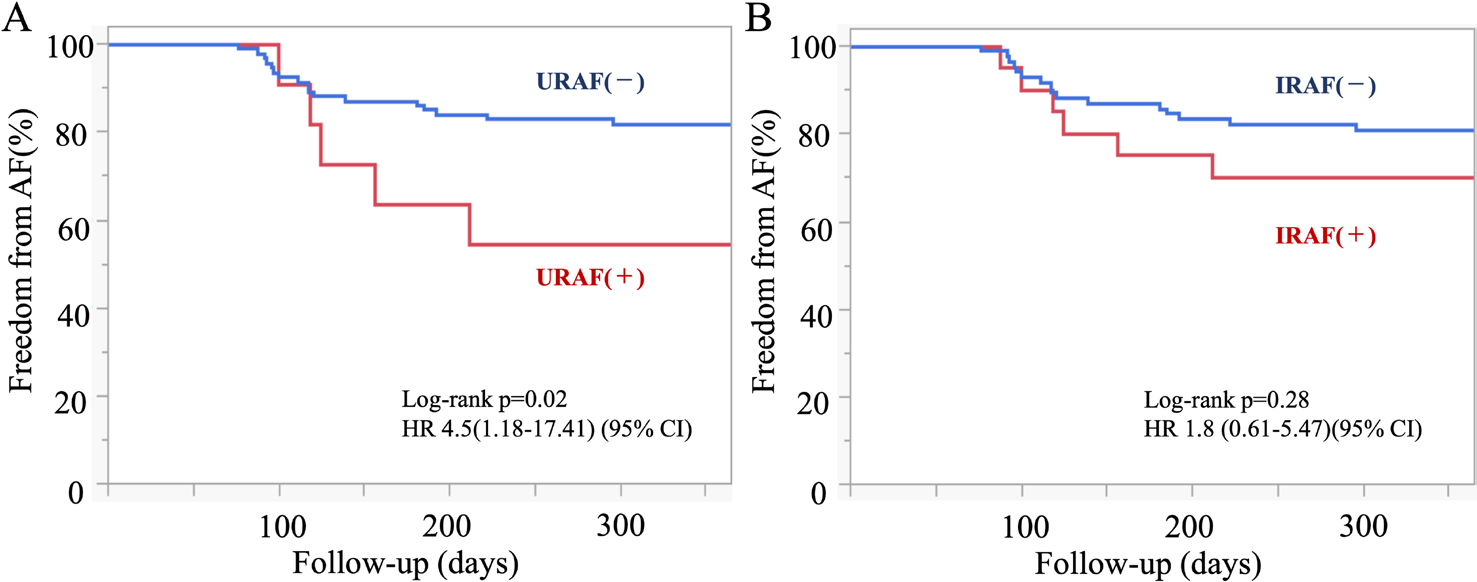
Kaplan–Meier curves for af recurrence. The recurrence‐free survival rate over 12 months is compared between patients with and without ultra‐early recurrence of atrial fibrillation (panel A) and with and without immediate recurrence of atrial fibrillation (panel B). Patients with URAF showed significantly higher recurrence rates than those without. URAF: Ultra‐early recurrence of atrial fibrillation; IRAF: Immediate recurrence of atrial fibrillation.

Kaplan–Meier analysis demonstrated that patients with URAF had a significantly higher recurrence rate compared to those without URAF (log‐rank *p* = 0.02; HR 4.5; 95% CI: 1.18–17.41; Figure 2A). In contrast, no significant difference in recurrence‐free survival was observed between patients with and without IRAF (log‐rank *p* = 0.28; HR 1.8; 95% CI: 0.61–5.47; Figure 2B).

Redo ablation findings are summarized in Table 6. Among the 22 patients with clinical recurrence, 14 underwent a second ablation. PV reconnection occurred in 2/3 (URAF+) versus 7/11 (URAF−) (Fisher's exact test, two‐sided, *p* = 0.76), and in 2/4 (IRAF+) versus 7/10 (IRAF−) (*p* = 0.45). Accordingly, PV reconnection rates did not differ significantly by either URAF or IRAF status in the redo cohort.

**TABLE 6:** PV Reconnection at Redo by URAF and IRAF Status.

Group (index)	Redo ablation, *n* (%)	PV reconnection, *n* (%)
A. By URAF status*		
URAF+	3/5 (60)	2/3 (67)
URAF−	11/17 (65)	7/11 (64)
Total	14/22 (64)	9/14 (64)
B. By IRAF status*		
IRAF+	4/6 (67)	2/4 (50)
IRAF−	10/16 (63)	7/10 (70)
Total	14/22 (64)	9/14 (64)

*Note:* *Fisher's exact (two‐sided) for URAF− vs. URAF+: *p* = 0.76 (for reference), IRAF− vs. IRAF+: *p* = 0.45 (for reference).

Abbreviations: IRAF, immediate recurrence of atrial fibrillation; PV, pulmonary vein; URAF, ultra‐early recurrence of atrial fibrillation.

## 4 Discussion

In this retrospective cohort study, we found that URAF—defined within 10 s after successful cardioversion under deep sedation—was associated with postablation recurrence in persistent AF. This suggests URAF may represent a promising intraprocedural marker associated with postablation recurrence in persistent AF. Importantly, this investigation was designed as an exploratory, hypothesis‐generating study. The findings provide mechanistic insights but are not yet ready to guide routine clinical practice. Consistent with the mechanistic rationale for the ≤ 10‐s window, broader cut‐offs (≤ 15, ≤ 30, ≤ 60 s) did not improve discrimination in our sensitivity analyses; therefore, we retain ≤ 10 s as the working definition, while acknowledging that these threshold analyses are exploratory and require prospective validation.

Among patients with persistent AF, 19.2% exhibited IRAF, and 50% of those met criteria for URAF. Notably, there were no significant differences in baseline characteristics—including age, sex, AF duration, LA diameter, or CHADS2 score—between patients with and without IRAF or URAF. This indicates that the occurrence of IRAF or URAF could not be predicted based on clinical background alone. Differences in procedural variables, such as the number of CV attempts and the performance of posterior LA wall ablation, were likely operator‐driven responses to IRAF or URAF rather than preprocedural predictors of these events.

URAF was associated with AF recurrence independently of conventional risk factors such as long‐standing persistent AF, left atrial diameter, and CHADS2 score [14, 15, 16]. In contrast, IRAF did not predict long‐term recurrence after ablation. This distinction is supported by earlier work from Timmermans et al., who reported that IRAF following internal atrial defibrillation did not predict future recurrence [17]. Furthermore, as IRAF is frequently triggered by foci originating from the pulmonary veins [12], its utility as a predictor of post‐PVI recurrence remains uncertain.

Inoue et al. reported that in patients with persistent AF, IRAF was associated with a high risk of recurrence unless the specific triggering focus was targeted during ablation [11]. According to previous studies, the mean time to IRAF was 20.2 ± 25.2 s [10] and 18.2 ± 23.0 s [8], closely matching our observation of 18.2 ± 19.6 s. Given that 50% of our patients with IRAF fulfilled criteria for URAF, it is plausible that many patients classified as having IRAF in Inoue's study may have actually experienced URAF, although this subset was not explicitly defined in their methodology. Notably, in our study, no ablation was performed against IRAF‐ or URAF‐related triggers, yet the presence of URAF prior to PVI independently predicted long‐term recurrence, underscoring its role as a substrate‐based procedural marker.

Unlike previous IRAF studies, our investigation controlled for autonomic tone by observing recurrence under deep sedation, suppressing sympathetic activity. These observations were made under deep sedation and during a 90‐s period of catheter immobility, reducing confounding from sympathetic activation and catheter manipulation; however, the underlying mechanism cannot be determined from the present data. Consistent with prior reports [11], recurrence was more frequent in patients with long‐standing persistent AF in our cohort. Structural and electrical remodeling are thought to influence ablation outcomes in persistent AF [4, 5]. URAF may indicate atrial substrate vulnerability; however, direct substrate characterization (e.g., low‐voltage mapping, imaging, or biomarkers) was not performed, and the mechanistic interpretation remains hypothesis‐generating.

In summary, in this retrospective single‐center cohort of persistent AF undergoing first‐time PVI, ultra‐early recurrence within 10 s after cardioversion under deep sedation was associated with 12‐month postablation recurrence. Taken together, these findings suggest that URAF may represent a promising intraprocedural marker associated with postablation recurrence in persistent AF. In sensitivity analyses, alternative thresholds (≤ 15, ≤ 30, ≤ 60 s) and conventional IRAF (≤ 90 s) did not outperform the prespecified ≤ 10‐s definition. These observations are exploratory; the underlying mechanism cannot be determined in the absence of direct substrate characterization, and prospective validation in larger, multicenter cohorts with extended or continuous rhythm monitoring is required.

## 5 Limitations

This single‐center retrospective study involved a relatively small sample size, which may limit generalizability. In particular, only 10 patients exhibited URAF, introducing potential statistical fragility and limiting reproducibility; therefore, all multivariable and subgroup findings should be interpreted as exploratory. We did not perform systematic electroanatomical substrate assessment, such as voltage mapping or MRI, limiting insight into the structural underpinnings of recurrence. Non‐PV triggers and other mechanisms were not systematically evaluated by protocol. Classifying patients who remained in sinus rhythm on arrival as IRAF−/URAF− may introduce selection bias. Furthermore, the reproducibility of recurrence timing after cardioversion was not evaluated, as repeated CV shocks were not ethically feasible due to the risk of myocardial injury [18]. Additionally, the 10‐s threshold for defining URAF was selected a priori to isolate the most immediate recurrences under parasympathetic‐dominant conditions. While conceptually grounded, this cut‐off warrants future prospective validation. AF recurrence was assessed by scheduled ECG and 24‐hour Holter monitoring, which likely underestimates the true recurrence burden—particularly asymptomatic episodes—relative to extended or continuous monitoring. Asymptomatic AF episodes were not systematically captured, potentially biasing recurrence classification toward nonrecurrence. Given the limited number of outcome events, the multivariable analysis should be interpreted as exploratory. Prospective, multicenter validation with extended or continuous rhythm monitoring is warranted to establish reproducibility and clinical utility. Finally, the reproducibility of URAF as a procedural biomarker was not tested prospectively, and whether the same patients would manifest URAF in repeated procedures remains unknown.

## 6 Conclusions

URAF may represent a promising intraprocedural marker associated with postablation recurrence in persistent AF, but prospective validation in larger cohorts is required.

## Conflicts of Interest

The authors declare no conflicts of interest.

## Data Availability

The data supporting the findings of this study are available from the corresponding author upon reasonable request.

## References

[1] D. Scherr , P. Khairy , S. Miyazaki , et al., “Five‐Year Outcome of Catheter Ablation of Persistent Atrial Fibrillation Using Termination of Atrial Fibrillation as a Procedural Endpoint,” Circulation. Arrhythmia and Electrophysiology 8 (2015): 18–24, 10.1161/CIRCEP.114.001943. 25528745

[2] N. Dagres , M. G. Bongiorni , T. B. Larsen , A. Hernandez‐Madrid , L. Pison , and C. Blomstrom‐Lundqvist , “Current Ablation Techniques for Persistent Atrial Fibrillation: Results of the European Heart Rhythm Association Survey,” Europace 17 (2015): 1596–1600, 10.1093/europace/euv323. 26498718

[3] M. Anselmino , M. Matta , F. D'Ascenzo , et al., “Catheter Ablation of Atrial Fibrillation in Patients With Left Ventricular Systolic Dysfunction: a Systematic Review and Meta‐Analysis,” Circulation. Arrhythmia and Electrophysiology 7 (2014): 1011–1018, 10.1161/CIRCEP.114.001938. 25262686

[4] M. A. Allessie , N. M. de Groot , R. P. Houben , et al., “Electropathological Substrate of Long‐Standing Persistent Atrial Fibrillation in Patients With Structural Heart Disease: Longitudinal Dissociation,” Circulation. Arrhythmia and Electrophysiology 3 (2010): 606–615, 10.1161/CIRCEP.109.910125. 20719881

[5] U. Schotten , J. Ausma , C. Stellbrink , et al., “Cellular Mechanisms of Depressed Atrial Contractility in Patients With Chronic Atrial Fibrillation,” Circulation 103 (2001): 691–698, 10.1161/01.cir.103.5.691. 11156881

[6] T. Kerola , T. A. Dewland , E. Vittinghoff , S. R. Heckbert , P. K. Stein , and G. M. Marcus , “Predictors of Atrial Ectopy and Their Relationship to Atrial Fibrillation Risk,” Europace 21 (2019): 864–870, 10.1093/europace/euz008. 30843034 PMC6545500

[7] D. Hamon , B. Courty , A. Leenhardt , et al., “Predictive Value of Premature Atrial Complex Characteristics in Pulmonary Vein Isolation for Patients With Paroxysmal Atrial Fibrillation,” Archives of Cardiovascular Diseases 114 (2021): 122–131, 10.1016/j.acvd.2020.09.001. 33153949

[8] H. Oral , M. Ozaydin , C. Sticherling , et al., “Effect of Atrial Fibrillation Duration on Probability of Immediate Recurrence After Transthoracic Cardioversion,” Journal of Cardiovascular Electrophysiology 14, no. 2 (2003): 182–185. 12693502

[9] B. Gorenek , G. Kudaiberdieva , Y. Cavusoglu , et al., “Immediate Recurrence of Atrial Fibrillation After Internal Cardioversion: Importance of Right Atrial Conduction Variations,” Journal of Electrocardiology 35, no. 4 (2002): 313–320, 10.1054/jelc.2002.36279. 12395358

[10] A. Chugh , M. Ozaydin , C. Scharf , et al., “Mechanism of Immediate Recurrences of Atrial Fibrillation After Restoration of Sinus Rhythm,” Pacing and Clinical Electrophysiology 27, no. 1 (2004): 77–82, 10.1111/j.1540-8159.2004.00389.x. PMID: 14720159. 14720159

[11] K. Inoue , T. Kurotobi , R. Kimura , et al., “Trigger‐Based Mechanism of the Persistence of Atrial Fibrillation and Its Impact on the Efficacy of Catheter Ablation,” Circulation Arrhythmia and Electrophysiology 5, no. 2 (2012): 295–301, 10.1161/CIRCEP.111.964080. 22042883

[12] C. A. Groh , M. Faulkner , S. Getabecha , et al., “Patient‐Reported Triggers of Paroxysmal Atrial Fibrillation,” Heart Rhythm 16 (2019): 996–1002, 10.1016/j.hrthm.2019.01.027. 30772533

[13] G. Boriani and M. Grimaldi , “2024 ESC Guidelines for the Management of Patients With Atrial Fibrillation: What's New and What Implications for Clinical Practice?,” G Ital Cardiol (Rome) 26 (2025): 3–13, 10.1714/4394.43951. 39714492

[14] A. Wokhlu , D. O. Hodge , K. H. Monahan , et al., “Long‐Term Outcome of Atrial Fibrillation Ablation: Impact and Predictors of Very Late Recurrence,” Journal of Cardiovascular Electrophysiology 21 (2010): 1071–1078, 10.1111/j.1540-8167.2010.01786.x. 20500237

[15] J. K. Park , J. Y. Lee , P. S. Yang , et al., “Good Responders to Catheter Ablation for Long‐Standing Persistent Atrial Fibrillation: Clinical and Genetic Characteristics,” Journal of Cardiology 69 (2017): 584–590, 10.1016/j.jjcc.2016.04.017. 27261248

[16] M. Kaneko , Y. Nagata , T. Nakamura , et al., “Geriatric Nutritional Risk Index as a Predictor of Arrhythmia Recurrence After Catheter Ablation of Atrial Fibrillation,” Nutrition, Metabolism, and Cardiovascular Diseases 31 (2021): 1798–1808, 10.1016/j.numecd.2021.03.004. 33985896

[17] C. Timmermans , L. M. Rodriguez , J. L. Smeets , and H. J. Wellens , “Immediate Reinitiation of Atrial Fibrillation Following Internal Atrial Defibrillation,” Journal of Cardiovascular Electrophysiology 9, no. 2 (1998): 122–128, 10.1111/j.1540-8167.1998.tb00893.x. 9511886

[18] G. Boriani , M. Biffi , V. Cervi , et al., “Evaluation of Myocardial Injury Following Repeated Internal Atrial Shocks by Monitoring Serum Cardiac Troponin I Levels,” Chest 118 (2000): 342–347, 10.1378/chest.118.2.342. 10936122

